# Regional Transport of PM_2.5_ from Coal-Fired Power Plants in the Fenwei Plain, China

**DOI:** 10.3390/ijerph20032170

**Published:** 2023-01-25

**Authors:** Pan Lu, Shunxi Deng, Guanghua Li, Abula Tuheti, Jiayao Liu

**Affiliations:** 1 School of Water and Environment, Chang’an University, Xi’an 710064, China; 2 School of Energy and Architecture, Xi’an Aeronautical Institute, Xi’an 710077, China; 3 School of Architectural Engineering, Chang’an University, Xi’an 710064, China; 4 Key Laboratory of Subsurface Hydrology and Ecological Effect in Arid Region of Ministry of Education, Chang’an University, Xi’an 710064, China

**Keywords:** PM_2.5_, coal-fired power plants, regional transport, CALPUFF

## Abstract

The Fenwei Plain (FWP) remains one of the worst PM_2.5_-polluted regions in China, although its air quality has improved in recent years. To evaluate the regional transport characteristics of PM_2.5_ emitted by coal-fired power plants in the FWP in wintertime, the primary PM_2.5_, SO_2_, and NOx emissions from coal-fired power plants with large units (≥300 MW) in 11 cities of the area in January 2019 were collected based on the Continuous Emission Monitoring System (CEMS). The spatial distribution and source contribution of primary and secondary PM_2.5_ concentrations were investigated using the Weather Research and Forecast (WRF) model and the California Puff (CALPUFF) model. The results showed that secondary PM_2.5_ was transported over a larger range than primary PM_2.5_ and that secondary nitrate was the main component of the total PM_2.5_ concentration, accounting for more than 70%. High concentrations of primary, secondary, and total PM_2.5_ mainly occurred in the Shaanxi region of the FWP, especially in Xianyang, where the PM_2.5_ concentrations were the highest among the 11 cities, even though its pollutant emissions were at moderate levels. The PM_2.5_ concentrations in Sanmenxia and Yuncheng primarily came from regional transport, accounting for 64% and 68%, respectively, while those in other cities were dominated by local emissions, accounting for more than 63%. The results may help to understand the regional transport characteristics of pollutants emitted from elevated point sources over a complex terrain.

## 1. Introduction

Currently, China is the largest coal producer and consumer in the world, and coal is the first fossil fuel in China’s energy structure [1,2]. In 2019, coal consumption in China was 4.02 billion tons, accounting for 57.7% of the total energy consumption, of which about half was used in coal-fired power plants [3]. Coal-fired power plants generate electricity by converting the thermal energy from high-temperature coal combustion into electrical energy. At the same time, a large amount of air pollutants are also produced during the combustion process and are discharged into the air through the stack. Previous studies have indicated that coal-fired power plants have become one of the important sources of PM_2.5_ emissions in Chinese cities [4,5,6]. In 2010 and 2014, coal-fired power plants were found to contribute 7.3% and 5.6% of China’s total PM_2.5_ emissions, respectively [7,8]. The PM_2.5_ from coal-fired power plants is mainly composed of water-soluble ions, carbon-containing components, heavy metal elements, and other chemical components, which can cause various adverse effects on human health, such as neurobehavioral symptoms [9,10], respiratory diseases [11,12,13], and cardiovascular diseases [14,15,16]. It can be seen that reducing the contribution of coal-fired power plants to near-surface PM_2.5_ concentrations is of great significance for improving the regional ambient air quality. Thus, it is necessary to conduct research on PM_2.5_ emissions from coal-fired power plants in regions with a large number of coal-fired power plants.

Pollutant emissions from tall stacks of coal-fired power plants belong to elevated point sources, which have drawn significant concern due to their large emissions and wide dispersion [17,18,19,20,21]. Currently, modeling studies have been widely used to estimate the spatial distribution and environmental contribution of PM_2.5_ concentrations from coal-fired power plants. The California Puff (CALPUFF) model was applied to calculate the contribution of the largest coal-fired power plant in Italy to its nearby particulate matter concentrations [22,23] and to estimate the dispersion characteristics of PM_2.5_ emitted by a coal-fired power plant in Spain [24]. In addition, the chemical conversion of gaseous precursors, such as sulfur dioxide (SO_2_) and nitrogen oxides (NOx), to secondary PM_2.5_ was also considered in the calculations of the CALPUFF model [25,26,27]. Changotra et al. used Industrial Source Complex-Short Term Version 3 (ISCST3) and the American Meteorological Society-Environmental Protection Agency Regulatory Model (AERMOD) to predict the ground-level concentrations of pollutants emitted by the Rajpura thermal power plant in India, respectively, and found that the overall results of AERMOD predictions were better than those of ISCST3 predictions [28]. However, in the validation of the simulation results of pollutant concentrations from power plants, the performance of the CALPUFF model was judged to be superior to that of AERMOD [29]. The Comprehensive Air Quality Model (CAMx) coupled with plume rise functions was used to simulate the dispersion of PM_2.5_ from 111 coal-fired power plants in India, and the analysis showed that the PM_2.5_ concentrations could be reduced by 30%-40% by eliminating the formation of secondary sulfates and nitrates [30]. Hu et al. adopted the Community Multiscale Air Quality (CMAQ) model to predict the impact of PM_2.5_ emissions from coal-fired power plants on air quality under five future power development scenarios in China [31]. Shi et al. used the CMAQ model to quantitatively estimate the contribution of different sectors of source emissions to ambient PM_2.5_ concentrations in China and found that power plants are also an important source of secondary inorganic aerosols [32]. The aforementioned studies have mainly focused on PM_2.5_ concentrations near coal-fired power plants. However, the regional transport of PM_2.5_ from coal-fired power plants has rarely been studied. Moreover, most of the existing research on the dispersion of PM_2.5_ emitted by coal-fired power plants in China has focused on the key areas of air pollution, such as the Beijing–Tianjin–Hebei region [4,26,33,34,35] or heavily polluted cities [6,36,37], while few studies have focused on the central and western regions of China, such as the Fenwei Plain. Therefore, this paper takes the Fenwei Plain as the research region and adopts the simulation method to study the regional transport of PM_2.5_ emitted by coal-fired power plants in this region.

The Fenwei Plain (FWP) is located in the Weihe River and Fenhe River basins, and many cities in the FWP experience serious air pollution problems, especially with high PM_2.5_ mass concentrations in winter. In January 2013, the average PM_2.5_ concentration during the haze period in Xi’an (a city in the FWP) was 345.1 µg/m^3^, substantially higher than that in Beijing (158.5 µg/m^3^) during the same period [38]. In 2018, 6 cities in the FWP were ranked among the top 20 cities in China with the worst PM_2.5_ pollution [39]. Therefore, together with the Beijing–Tianjin–Hebei region and the Yangtze River Delta region, the FWP was designated as one of the three key regions for the “Blue Sky Protection Campaign” (2018–2020) in China in 2018 [40]. From January to February 2019, the average PM_2.5_ concentrations in the Yuncheng–Linfen region and the Xianyang–Xi’an region of the FWP were 135.3 and 137.0 µg/m^3^, respectively [41]. The severe air pollution in the FWP is directly related to its massive anthropogenic source emissions. The Fenwei Plain is an important coal-producing area, and coal accounts for 90% of its energy consumption, so the pollution caused by coal combustion emissions is also obvious [42]. It has been reported that the contribution of coal combustion to the PM_2.5_ concentration in Xi’an reached 20.6% in the winter of 2019 [41]. As a key coal-consuming industry in the FWP, coal-fired power plants are one of the important industries responsible for regional haze pollution. However, few studies have specifically targeted the concentration distribution and regional transport of primary and secondary PM_2.5_ emitted by the coal-fired power plants in cities in the FWP.

In this study, based on the Continuous Emission Monitoring System (CEMS), we collected primary PM_2.5_, SO_2_, and NOx emissions from coal-fired power plants in the FWP in January 2019. Next, we applied the Weather Research and Forecast (WRF) model and the CALPUFF model to simulate the meteorological field as well as the spatial distribution of primary PM_2.5_, secondary sulfate (SO_4_^2−^), secondary nitrate (NO_3_^−^), and total PM_2.5_ concentrations emitted by coal-fired power plants. Finally, the contribution of local emissions and regional transport to the PM_2.5_ concentrations in cities in the FWP was calculated. The results may be useful for the formulation of PM_2.5_ concentration reduction strategies in the FWP.

## 2. Materials and Methods

### 2.1. Study Area

**Figure 1:**
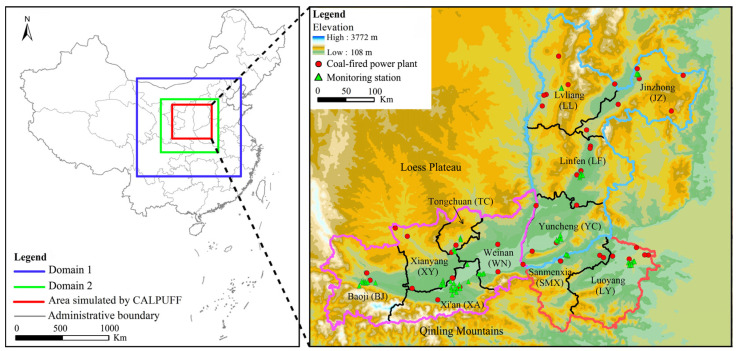
The location and topography of the Fenwei Plain (FWP) and the simulated domains.

The location of the FWP is shown in Figure 1. The FWP consists of the Fenhe Plain, the Guanzhong Plain, and its surrounding loess terraces. It is in the shape of a long and narrow crescent, surrounded by the Qinling Mountains in the south and the Loess Plateau in the north. The FWP is the fourth-largest plain in China, measuring 760 km from east to west and 40–100 km from north to south, with a total area of about 70,000 km^2^. The altitude ranges from 323 m to 800 m. The FWP includes 11 prefecture-level cities, namely Baoji (BJ), Xi’an (XA), Xianyang (XY), Tongchuan (TC), and Weinan (WN) in Shaanxi Province; Sanmenxia (SMX) and Luoyang (LY) in Henan Province; and Yuncheng (YC), Linfen (LF), Jinzhong (JZ), and Lvliang (LL) in Shanxi Province. In 2021, the residential population stood at 55.54 million.

### 2.2. Data Source

#### 2.2.1. Emission Data

**Figure 2:**
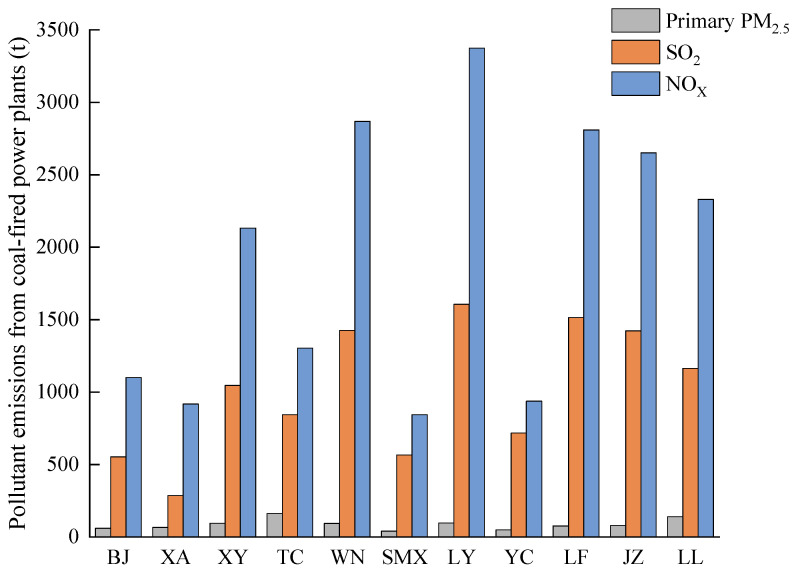
Pollutant emissions from coal-fired power plants with large units (≥300 MW) in 11 cities in the FWP in 2019.

In the research on pollutant emissions of the coal-fired power plants in Shaanxi Province by Xu et al. [43], the results showed that although the number of coal-fired power plants with large units (≥300 MW) was small, they contributed more than 70% of the total pollutant emissions from coal-fired power plants in the region. Therefore, this paper takes the coal-fired power plants with large units in the FWP as the research object. By the end of 2019, there were 47 coal-fired power plants with large units in the FWP, including 15 in the Shaanxi region, 12 in the Henan region, and 20 in the Shanxi region (see Figure 1). These coal-fired power plants used lignite as fuel and were equipped with flue gas desulfurization, denitrification, and dust removal facilities. Based on the data from the Continuous Emission Monitoring System (CEMS) of flue gas, and with reference to the data from the National Emission Permit Information Management Platform (NEPIMP; http://permit.mee.gov.cn, accessed on 4 March 2022) and the Environmental Law Enforcement Department (ELED), we obtained pollutant emissions from the coal-fired power plants in the FWP. Table 1 shows the primary PM_2.5_, SO_2_, and NO_X_ emissions in each region of the FWP in 2019. Pollutant emissions from coal-fired power plants in 11 cities are shown in Figure 2. The emissions in the Henan region were much smaller than those in Shaanxi and Shanxi regions. LY had the largest SO_2_ and NOx emissions. The emissions of the three pollutants in XA, SMX, and YC were relatively small.

#### 2.2.2. Meteorological Data

The initial meteorological data used by the WRF model were derived from the Final Operational Global Analysis (FNL) data of the National Center for Environmental Prediction (NCEP), and these data were at 6 h intervals with a horizontal resolution of 1° × 1° (https://rda.ucar.edu/datasets/ds083.2/, accessed on 17 March 2022). To verify the reliability of the meteorological simulation results, we collected the actual hourly meteorological data recorded by meteorological observation stations from the National Meteorological Information Center (http://data.cma.cn, accessed on 21 April 2022).

### 2.3. Air Quality Model

Air quality models have proven useful in calculating the transport of emitted air pollutants and in developing air quality control policies [44,45]. At present, common air quality models include AERMOD, CALPUFF, CAMx, and CMAQ, which have been widely used in various case studies [46,47,48,49,50]. Compared with other models, CALPUFF has the characteristics of open source, wide applicability, and good prediction performance. It has been adopted by the US Environment Protection Agency (US EPA) and the Ministry of Ecology Environment of the People’s Republic of China (MEEC) as the preferred model for assessing the long-range (more than 50 km) transport of pollutants in complex terrains [51,52]. Therefore, the WRF/CALPUFF modeling system was used in this study to simulate the transport, transformation, and removal of pollutants from coal-fired power plants in the Fenwei Plain.

The WRF model is the latest-generation mesoscale numerical weather prediction model, which can be widely used for meteorological research and numerical weather prediction from a few kilometers to thousands of kilometers [53,54]. In this paper, the WRF model (version 3.9.1) was used to provide a large-scale initial guess meteorological field for the CALPUFF model. The WRF model was configured with two nested domains (see Figure 1), both centered at 108.95° E, 34.26° N, and with 32 vertical layers up to 50 hPa. The outer domain 1 (D1) had a horizontal resolution of 18 km and a grid number of 100 × 92. The inner domain 2 (D2) covered most of Shaanxi, Henan, and Shanxi Provinces and their adjacent areas. The horizontal resolution of D2 was 6 km, and the number of grids was 180 × 156. The main physical parameterization schemes for the WRF model are given in Table 2.

CALPUFF is a non-steady-state, multi-layer Gaussian puff dispersion model that can simulate the transport of multiple air pollutants by considering the effects of temporally and spatially varying meteorological conditions in three dimensions. The CALPUFF model system mainly consists of three components: a diagnostic meteorological model (CALMET), an air quality dispersion model (CALPUFF), and a post-processing package (CALPOST). CALMET was used to adjust the meteorological fields from the WRF model to reflect the terrain and land use. In this study, the terrain and land use data were derived from the United States Geological Survey (USGS) data. CALMET was configured with a domain covering the entire Fenwei Plain (see Figure 1), with a horizontal resolution of 3 km and a grid number of 210 × 182. In the vertical dimension, the 10 height layers incorporated in this model were 20 m, 40 m, 80 m, 100 m, 300 m, 640 m, 1000 m, 2000 m, 3000 m, and 4000 m. The main parameter settings for CALPUFF are given in Table 3. The MESOPPUFF II chemical transformation scheme, designated as the preferred option by the US EPA, was used to simulate the conversion of the emitted gas pollutants SO_2_ and NOx to secondary SO_4_^2−^ and NO_3_^−^. These gas–particle chemical reactions involved six pollutants (SO_2_, NOx, SO_4_^2−^, NO_3_^−^, hydrogen nitrate (HNO_3_), and PM_2.5_) and required ambient background ammonia (NH_3_) and ozone (O_3_) concentrations. The hourly average concentrations of NH_3_ and O_3_ recorded by 62 state-controlled air quality–monitoring stations in the FWP (see Figure 1) were entered into the model as background concentrations. The SO_4_^2−^ and NO_3_^−^ concentrations calculated from the MESOPUFF II model were multiplied by coefficients of 1.374 and 1.29, respectively, to obtain the concentrations of ammonium sulfate ((NH_4_)_2_SO_4_) and ammonium nitrate (NH_4_NO_3_) [52,55]. In this study, the secondary PM_2.5_ concentration was calculated by adding the concentrations of 1.374SO_4_^2−^ and 1.29NO_3_^−^. Therefore, the sum of the primary PM_2.5_, 1.374SO_4_^2−^, and 1.29NO_3_^−^ concentrations was the total PM_2.5_ concentration. In addition, both dry and wet deposition of PM_2.5_ was also considered in this study. In the CALPUFF model, the emission source inventories of all coal-fired power plants were input as elevated point sources.

## 3. Results and Discussion

### 3.1. Verification of Meteorological Simulations

**Figure 3:**
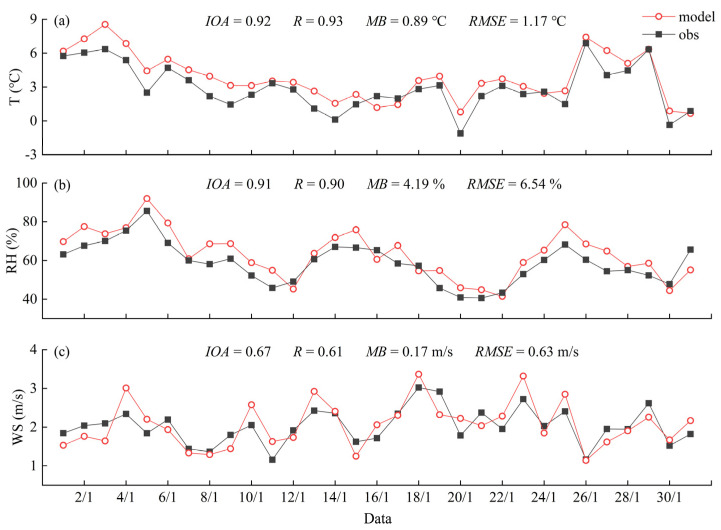
The time series of daily simulated and observed average temperatures (**a**), average relative humidity (**b**), and average wind speeds (**c**) at meteorological sites in Xi’an in January 2019.

**Figure 4:**
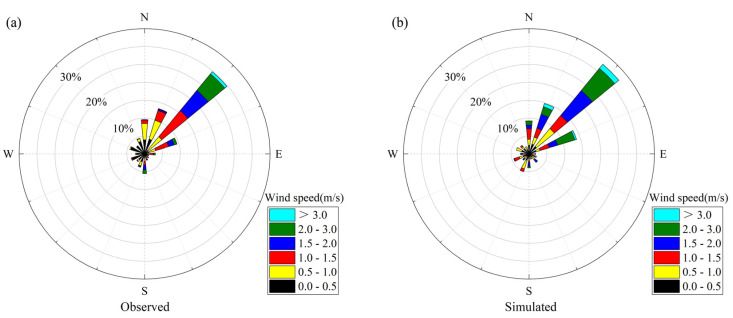
The wind roses obtained by observation (**a**) and simulation (**b**) at the Caotang observation site in January 2019.

To evaluate the validity of the WRF/CALMET model, the simulated results were compared with the observational data from nine meteorological sites in XA. The time series of daily simulated and observed average temperatures (T) at a 2 m height, average relative humidity (RH) at a 2 m height, and wind speeds (WS) at a 10 m height among all sites in January 2019 are shown in Figure 3. Figure 4 shows the wind rose plots at the Caotang observation site in XA. In addition, the four statistical indicators (the index of agreement (IOA), the Pearson correlation coefficient (R), the mean bias (MB), and the root mean square error (RMSE)) were used to verify the model performance by comparing the hourly simulated values (T, RH, and WS) with the observed values (see Figure 3).

In Figure 3, the simulated mean values of T, RH, and WS were 3.86 °C, 63.17%, and 2.07 m/s, respectively, while their observed mean values were 2.97 °C, 58.69%, and 2.03 m/s, respectively. Obviously, T and RH were clearly overestimated, while WS was relatively consistent. As can be seen in Figure 4, the prevailing wind direction in January 2019 was northeast wind, and the simulation results were relatively accurate. For statistical indicators, the simulation results have certain credibility when the IOA is greater than 0.5 [56,57]. The averaged IOA values were 0.92 for T, 0.91 for RH, and 0.67 for WS, indicating a high correlation and agreement between the simulated and observed values. The averaged R values of T, RH, and WS were 0.93, 0.90, and 0.61, respectively, and all R values were close to 1, which indicates a good agreement between the calculated results and the observations. The MB reflects the systematic error, the RMSE represents the square root of the mean squared error, and the closer their values are to 0, the more accurate the simulation results are [58]. The MB and RMSE values were only 0.89 °C and 1.17 °C for T, 4.19% and 6.54% for RH, and 0.17 m/s and 0.63 m/s for WS, respectively. Based on these results, the meteorological simulation of the WRF/CALMET model showed sufficient reliability and precision, which laid the foundation for the CALPUFF model to accurately predict the transport of pollutants.

### 3.2. Spatial Distribution of PM_2.5_ Concentrations

**Figure 5:**
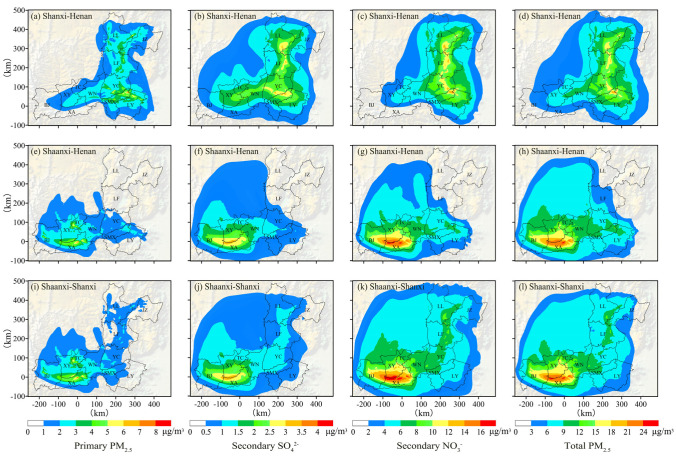
The spatial distributions of the maximum hourly concentrations of primary PM_2.5_, secondary SO_4_^2−^, secondary NO_3_^−^, and total PM_2.5_ from the Shanxi–Henan group (**a**–**d**), the Shaanxi–Henan group (**e**–**h**), and the Shaanxi–Shanxi group (**i**–**l**) in January 2019.

To determine the extent to which one region is affected by the transport of pollutants from the coal-fired power plants in other regions in the FWP, the district-based method was used to divide pollutant sources into three groups: Shanxi–Henan, Shaanxi–Henan, and Shaanxi–Shanxi. To define the peak of the spatial distributions of the PM_2.5_ concentration in January, the maximum value of hourly concentration in the whole simulation period was adopted in this section. Figure 5 presents the spatial distributions of the maximum hourly concentrations of primary PM_2.5_, secondary SO_4_^2−^, secondary NO_3_^−^, and total PM_2.5_ (primary PM_2.5_ + secondary PM_2.5_) from different groups during the simulation period.

As shown in the figure, the higher concentrations of primary PM_2.5_ were clearly concentrated at the locations of coal-fired power plants. Meanwhile, under the influence of the dominant northeasterly wind, the primary PM_2.5_ concentrations rapidly decreased along the downwind direction and dispersed to the surrounding areas. Compared to primary PM_2.5_, secondary SO_4_^2−^ and NO_3_^−^ had a larger concentration distribution range, mainly because the gaseous SO_2_ and NOx emitted by tall stacks were more easily transported over long distances. High concentrations of secondary SO_4_^2−^ and NO_3_^−^ tended to appear in the low-lying areas of the FWP, especially in the Shaanxi region, where the pollutants were not easily dispersed due to the influence of the Loess Plateau in the north and the Qinling Mountains in the south. Overall, the concentrations of secondary NO_3_^−^ generated from the three groups were much greater than those of secondary SO_4_^2−^. The main reason for this was that the NOx emissions from all coal-fired power plants were much greater than the SO_2_ emissions. Furthermore, previous studies have shown that the ambient temperature below 15 °C in winter is more conducive to the conversion of gaseous NOx to particulate NO_3_^−^ [59,60,61], while the average temperature during the concentration calculation period in this study was only 3.86 °C. Studies have also indicated that in the haze pollution stage of ambient PM_2.5_ concentration < 150 µg/m^3^, the relative humidity value exhibits a positive relationship with the secondary NO_3_^−^ concentration [62,63], and the average value of the simulated RH in this paper was 63.17%. For total PM_2.5_, the spatial distribution trends of its concentrations were similar to those of secondary NO_3_^−^ concentrations. In Figure 5l, the maximum concentration of total PM_2.5_ from the Shaanxi–Shanxi group reached 26.37 µg/m^3^, which is consistent with the results obtained by Chen et al. using a chemical transport model and a receptor model, who found that the contribution of coal-fired power plants to the near-surface PM_2.5_ concentration during the severe pollution period is at least 10 µg/m^3^ [4].

In the regional transport of primary, secondary, and total PM_2.5_ in the FWP, power plant emissions from three different groups all had the greatest impact on their nearest cities, mainly WN, YC, and SMX. Owing to the topography and prevailing wind direction in winter, a large area of the Shaanxi region was affected by the emissions from the Shanxi–Henan group, and the cities involved mainly included WN, TC, and XY. In addition, the PM_2.5_ emitted by the coal-fired power plants in the FWP could be transported across its boundaries to adjacent areas, especially to the Loess Plateau in the northwest.

**Figure 6:**
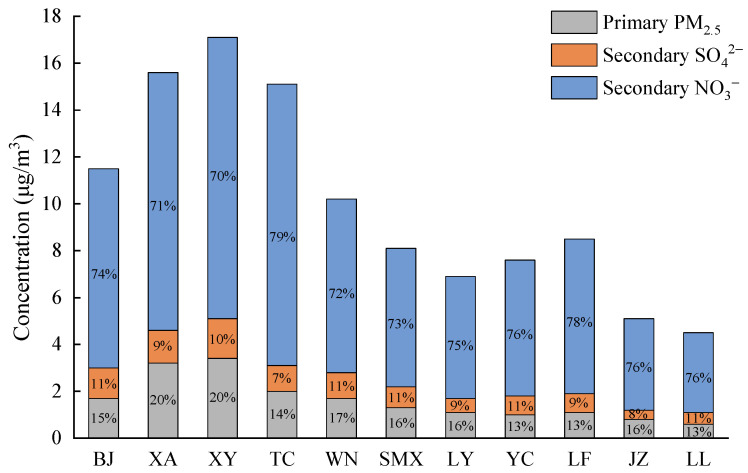
The hourly average of total PM_2.5_ concentrations and the proportion of primary and secondary PM_2.5_ in cities in the FWP.

In this study, the primary and secondary PM_2.5_ concentrations at the locations of the state-controlled air quality–monitoring stations (see Figure 1) in the concentration fields generated by coal-fired power plants were extracted and used to represent and analyze the PM_2.5_ concentrations from coal-fired power plants for each city. To reflect the composition of the total PM_2.5_ concentration in each city during the whole calculation period, the average value of the PM_2.5_ concentration over all hours, namely the hourly average concentration, was used for analysis. Figure 6 shows the hourly average PM_2.5_ concentrations for each city. The hourly average total PM_2.5_ concentration in the whole Fenwei Plain was 10.28 µg m^−3^. Higher concentrations of primary, secondary, and total PM_2.5_ all appeared in the cities of the Shaanxi region, and all reached their maximum in XY. However, except for WN, the concentrations of primary PM_2.5_, SO_2_, and NOx in other cities in the Shaanxi region were not large (see Figure 2). The PM_2.5_ concentrations in JZ and LL were lower than those in other cities. For all cities, the secondary NO_3_^−^ concentration was much greater than the primary PM_2.5_ concentration and the primary PM_2.5_ concentration was greater than the secondary SO_4_^2−^ concentration. The proportions of primary PM_2.5_, secondary SO_4_^2−^, and secondary NO_3_^−^ in the total PM_2.5_ concentrations in all cities were 13–20%, 7–11%, and 70–79%, respectively, which is in accordance with the research of Guttikunda and Jawahar on coal-fired power plants in India using the CAMx model [30].

### 3.3. Contribution of Local Emissions and Regional Transport to PM_2.5_ Concentration

**Figure 7:**
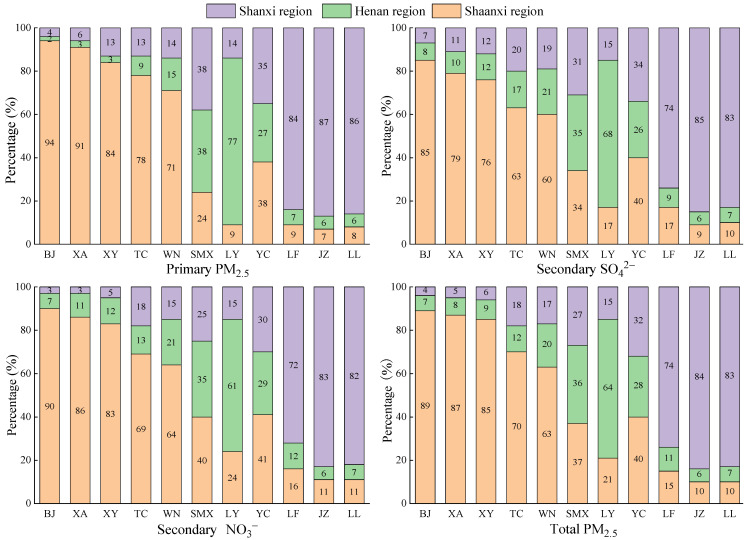
Concentration contributions of primary PM_2.5_, secondary SO_4_^2−^, secondary NO_3_^−^, and total PM_2.5_ in all cities in the FWP in January 2019.

To calculate the contribution of local emissions and regional transport to the PM_2.5_ concentrations of each city in the FWP, the hourly average PM_2.5_ concentration fields caused by the emissions from the Shaanxi, Henan, and Shanxi regions were simulated separately. Figure 7 shows the contribution of local emissions and regional transport to the hourly average concentrations of primary, secondary, and total PM_2.5_ in all cities in January 2019.

Overall, the proportion of local emissions to primary PM_2.5_ concentrations in all cities was greater than that of local emissions to secondary SO_4_^2−^ and secondary NO_3_^−^ concentrations, which indicated that the secondary PM_2.5_ generated through air–particle conversion is more easily dispersed than the primary PM_2.5_ directly emitted. Apparently, except for SMX and YC, local emissions in other cities were the dominant contributors to their near-surface primary, secondary, and total PM_2.5_ concentrations, contributing more than 60%, which was close to the findings of Yang et al. [64]. For the Shaanxi region, the contributions of regional transport to PM_2.5_ concentrations in all cities followed the order of WN > TC > XY > XA > BJ. In particular, the contribution of regional transport to the secondary SO_4_^2−^ concentrations in WN accounted for 40%. The contribution from the Shanxi region to the PM_2.5_ concentrations in TC was greater than that from the Henan region, while these contributions to WN were the opposite. For the Henan region, the PM_2.5_ concentrations in SMX mostly came from regional transport, accounting for 62–65%. The transport from the Shanxi region contributed higher to its primary PM_2.5_ concentrations than that from the Shaanxi region. However, the transport contribution from the Shaanxi region to its secondary and total PM_2.5_ concentrations was greater than that from the Shanxi region. For the Shanxi region, the PM_2.5_ concentrations in YC were mainly derived from regional transport, with local emissions, transport from the Shaanxi region, and transport from the Shanxi region accounting for 30–35%, 38–41%, and 26–29%, respectively. With regard to LF, JZ, and LL, the contributions of regional transport to their PM_2.5_ concentrations were similar.

## 4. Conclusions

In this study, we collected the pollutant emissions (primary PM_2.5_, SO_2_, and NOx) from coal-fired power plants in the FWP in January 2019, based on the CEMS data. The WRF/CALPUFF modeling system was used to simulate the spatial distribution of primary PM_2.5_, secondary SO_4_^2−^, secondary NO_3_^−^, and total PM_2.5_ concentrations from the Shanxi–Henan group, the Shaanxi–Henan group, and the Shaanxi–Shanxi group. The impact of local emissions and regional transport on PM_2.5_ concentrations was analyzed for 11 cities in the FWP, considering only the coal-fired power plant sources. In addition, to evaluate the performance of the WRF/CALMET model, the meteorological simulation results were compared with the observational data by calculating four statistical indicators.

The statistical results indicated that the WRF/CALMET model could provide reliable meteorological inputs for driving the CALPUFF model. In the distribution of PM_2.5_ concentrations, the higher primary PM_2.5_ concentrations were obviously concentrated at the locations of coal-fired power plants, while the higher secondary SO_4_^2−^ and NO_3_^−^ concentrations were distributed over a relatively wide range. Emissions from all three groups had the greatest impact on the PM_2.5_ concentrations in their neighboring cities. Due to the terrain and prevailing wind direction in winter, both primary and secondary PM_2.5_ in the Shaanxi region were less easily dispersed than those in Henan and Shanxi regions. Especially in XY in the Shaanxi region, although its pollutant emissions were at a moderate level among the 11 cities, its primary, secondary, and total PM_2.5_ concentrations were all the highest. In the total PM_2.5_ concentration of all cities in the FWP, the secondary NO_3_^−^ accounted for the largest proportion, reaching more than 70%. In the calculation of PM_2.5_ concentration contribution, the local emissions of other cities, except for SMX and YC, were the main contributors to their near-surface primary, secondary, and total PM_2.5_ concentrations, with the contribution rates exceeding 60%. The regional transport from Shaanxi and Shanxi regions accounted for 37% and 27% of the total PM_2.5_ concentration in SMX, respectively, and the regional transport from Shaanxi and Henan regions accounted for 40% and 28% of the total PM_2.5_ concentration in YC, respectively. The findings of this study can provide a valuable reference for the regional joint prevention and control of haze pollution in the Fenwei Plain in winter.

## Figures and Tables

**Table 1:** Primary PM_2.5_, SO_2_, and NO_X_ emissions in each region of the FWP in 2019 (t).

Region	Primary PM_2.5_	SO_2_	NOx
Shaanxi	476.47	4154.67	8321.46
Henan	136.80	2172.29	4218.63
Shanxi	345.38	4818.52	8728.74

**Table 2:** Main physical parameterization schemes for the WRF model.

Parameterization Scheme	Scheme Name
Solar radiation scheme	Dudhia scheme
Longwave radiation scheme	Rapid Radiative Transfer Model (RRTM)
Land surface process	Noah Land Surface Model
Boundary layer scheme	Asymmetric Convective Model 2.0 (ACM2)
Microphysics scheme	WRF Single-Moment 6-class (WSM6) scheme
Cumulus convection scheme	Kain–Fritsch scheme

**Table 3:** Main parameters used in CALPUFF.

Model Parameter	Parameter Settings
Model version	6.42
Domain size	630 km × 546 km
Map projection	Lambert conic conformal
Plume rise	Transitional plume rise modeled/partial plume penetration modeled: point sources
Plume element modeled	Puff
Chemical transformation method	MESOPUFF II
Dispersion option	Turbulence computed from micrometeorology
Terrain adjustment	ISC terrain adjustment scheme
Deposition	Vertical Structure and Mass Depletion/Resistance Deposition Model
Initial and boundary conditions	Default

## Data Availability

All data are presented within the article.
